# Turning Simulation into Estimation: Generalized Exchange Algorithms for Exponential Family Models

**DOI:** 10.1371/journal.pone.0169787

**Published:** 2017-01-11

**Authors:** Maarten Marsman, Gunter Maris, Timo Bechger, Cees Glas

**Affiliations:** 1 Department of Psychology, University of Amsterdam, Amsterdam, the Netherlands; 2 Psychometric Research Center, Cito, Arnhem, the Netherlands; 3 Department of Research Methodology, Measurement and Data Analysis, University of Twente, Enschede, the Netherlands; Banner Alzheimer’s Institute, UNITED STATES

## Abstract

The Single Variable Exchange algorithm is based on a simple idea; any model that can be simulated can be estimated by producing draws from the posterior distribution. We build on this simple idea by framing the Exchange algorithm as a mixture of Metropolis transition kernels and propose strategies that automatically select the more efficient transition kernels. In this manner we achieve significant improvements in convergence rate and autocorrelation of the Markov chain without relying on more than being able to simulate from the model. Our focus will be on statistical models in the Exponential Family and use two simple models from educational measurement to illustrate the contribution.

## 1 Introduction

In Bayesian statistical modeling, researchers formalize their substantive theories in a statistical model *π*(*x* ∣ *θ*) for the distribution of the data *x* and a prior distribution *π*(*θ*) for the parameter *θ*. Together, the statistical model and the prior distribution lead to the posterior distribution *π*(*θ* ∣ *x*). The desired posterior distribution is often intractable, and simulating draws from such posterior distributions can be a difficult task. However, simulating data from the model is often simple: first generate a parameter value θ* from the prior distribution *π*(*θ*) and then generate data x* from the statistical model *π*(*x* ∣ θ*). From this composite sampling scheme we obtain draws from the joint distribution [1]:

π(x,θ)=π(θ)π(x∣θ)=π(x)π(θ∣x), (1)

showing that the generated value θ* is also a draw from the posterior distribution *π*(θ* ∣ x*) ∝ *π*(x* ∣ θ*)*π*(θ*) [2]. The posterior distribution *π*(θ* ∣ x*) is equal to the desired posterior distribution *π*(*θ* ∣ *x*) only if the generated data x* are equal to the observed data *x*. Here we improve on an algorithm that uses the composite data sampling scheme to obtain draws from the posterior distribution *π*(*θ* ∣ *x*) to be used in a wide range of settings.

To simulate draws from the desired posterior *π*(*θ* ∣ *x*), Murray, Ghahramani and MacKay [3] cleverly utilized the posteriors *π*(*θ* ∣ x*) as proposal distributions in an independence chain Metropolis algorithm [4]. This Metropolis algorithm operates by constructing a discrete-time Markov chain with state space Ω_*θ*_ that has the desired posterior *π*(*θ* ∣ *x*) as invariant distribution [5], and can be characterized as follows:

$$\Theta_{t+1}=\left\{\begin{array}{cc} \Theta^{*}=\theta^{*} & \text{with}\;\text{probability}\;\phi (\theta^{\prime }\text{,}\;\theta^{*}|x\text{,}\;x^{*})\\ \Theta_{t}=\theta^{\prime } & \text{with}\;\text{probability}\;1-\phi (\theta^{\prime }\text{,}\;\theta^{*}|x\text{,}\;x^{*}) \end{array}\right.∼\Theta_{t}∼\pi \left(\cdot |x\right),$$

where the state *θ*′ of the chain at a time *t* is a draw from the invariant distribution *π*(*θ* ∣ *x*), the proposed value θ* is a draw from the proposal distribution *π*(*θ* ∣ x*), with *θ*′ and θ* ∈ Ω_*θ*_. The proposed point θ* is accepted if *U* < *ϕ*, with *U* ∼ Uniform(0, 1) and:

$$\phi \left(\theta^{\prime }\text{,}\;\theta^{*}|x\text{,}\;x^{*}\right)=\min\left(1,\frac{\pi \left(\theta^{*}|x\right)\pi \left(\theta^{\prime }|x^{*}\right)}{\pi \left(\theta^{\prime }|x\right)\pi \left(\theta^{*}|x^{*}\right)}\right)=\min\left(1,\frac{\pi \left(x|\theta^{*}\right)\pi \left(x^{*}|\theta^{\prime }\right)}{\pi \left(x|\theta^{\prime }\right)\pi \left(x^{*}|\theta^{*}\right)}\right).$$ (2)

Note that the proposed value θ* is always accepted, i.e., *ϕ*(*θ*′, θ* ∣ *x*, x*) = 1, if the proposed setting *π*(*x* ∣ θ*)*π*(x* ∣ *θ*′) is more likely than the current setting *π*(*x* ∣ *θ*′)*π*(x* ∣ θ*), otherwise it is accepted with a probability proportional to the ratio of likelihoods in the proposed and current setting. This is known as the Single-Variable-Exchange (SVE) algorithm [3] and makes simulating draws from a posterior distribution as simple as simulating data.

Although the SVE algorithm presents a practical and simple solution to sampling from intractable posterior distributions, its application and development has focused exclusively on statistical models *π*(*x* ∣ *θ*) with computationally intractable normalizing constants [6]. In fact, the SVE algorithm was originally developed for statistical models in the Exponential Family [7–9]; i.e., models that can be written as:

$$\pi \left(x|\theta \right)=\frac{1}{Z_{\theta }}h\left(x\right)\exp\left\{\theta \cdot t\left(x\right)\right\},$$

where *t*(*x*) is a (vector of) statistic(s) sufficient for *θ* and *Z*_*θ*_ a normalizing constant. Observe that for models in the Exponential Family, the acceptance probability is of a particular simple form:

$$\phi \left(\theta^{\prime }\text{,}\;\theta^{*}|t\left(x\right)\text{,}\;t\left(x^{*}\right)\right)=\min\left(1\text{,}\;\exp\{\;\left(\theta^{*}-\theta^{\prime }\right)\cdot \left(t\left(x\right)-t\left(x^{*}\right)\right)\;\}\;\right),$$ (3)

and does not involve the normalizing constant *Z*_*θ*_; making the SVE algorithm a practical tool for Bayesian inference of models where *Z*_*θ*_ is intractable, such as Exponential Random Graphs [10, 11] and Markov Random Fields [12, 13]. Despite the simplicity with which the SVE algorithm operates, especially for models in the Exponential Family (e.g., generalized linear models), its application to tractable statistical models *π*(*x* ∣ *θ*) has been completely unexplored.

Simple implementations do not guarantee efficient Markov chains, and in practice we often see that the SVE algorithm operates with low efficiency; requiring many thousands of iterations to obtain accurate estimates and wasting expensive computations on rejected proposals. This inefficiency results from generating data sets x* that are unlikely to occur under the current setting *θ*′ (or *x* under θ*); i.e. statistics *t*(x*) that are far from *t*(*x*) in Eq (3). To this aim, several approaches have been proposed that replace the simple proposal generating mechanism with more elaborate schemes, using, for instance, random walks [3], parallel tempering [3], population Markov chain Monte Carlo methods [10, 14], Langevine diffusions [15], or delayed rejection [16]. Although these approaches improve the statistical efficiency, they often fail to generalize the simple implementation of the original SVE algorithm.

Consequently, our goals are two-fold. Our primary goal is to show several developments that improve the statistical efficiency of the original SVE algorithm and result from reformulating it as an instance of what Tierney refers to as a mixture of Metropolis kernels [4, 17] in Section 2.1. Our efforts focus on simultaneously sampling from the posterior distribution of a large number of latent variables (e.g., random effects in generalized linear mixed models or Bayesian hierarchical models) in Sections 3.2 and 3.3, and on sampling from highly concentrated posterior distributions in Sections 3.1, 3.4 and 3.5. The strategies that we present improve their efficiency as the sample size grows (driving the autocorrelation to zero), and allow the utilization of the cheap parallelism that is available in modern day computing [18]. A simple Exponential Family model serves to illustrate the development.

**Fig 1:**
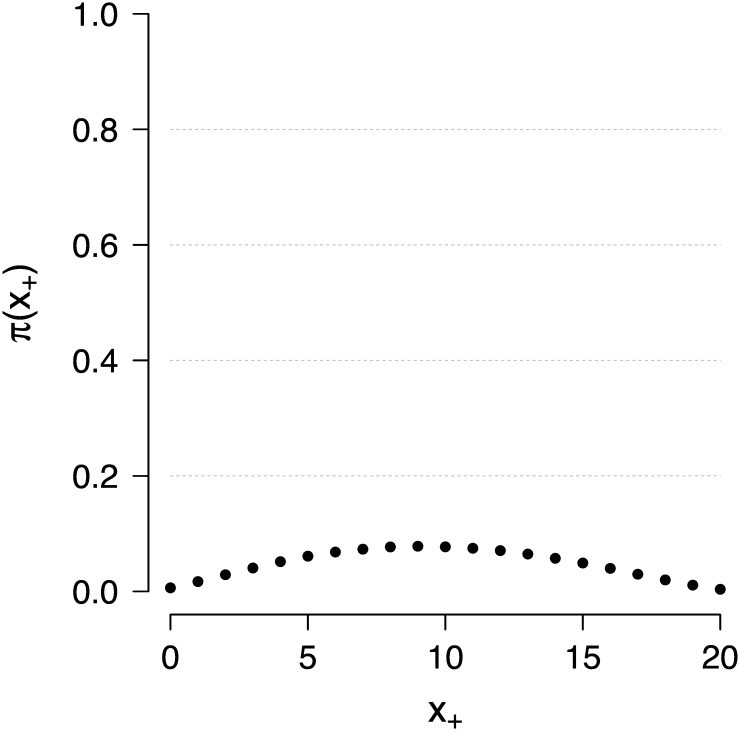
A mixing distribution $\pi (x_{+}^{*})$ for the original SVE. The distribution of transition kernels, i.e., $\pi (x_{+}^{*})$, for the original SVE algorithm in the Rasch model example with *k* = 20 items. In this example the average acceptance rate for sampling from the posterior *π*(*θ* ∣ *x*_+_ = 9) was approximately 37% (see S1 Script).

**Fig 2:**
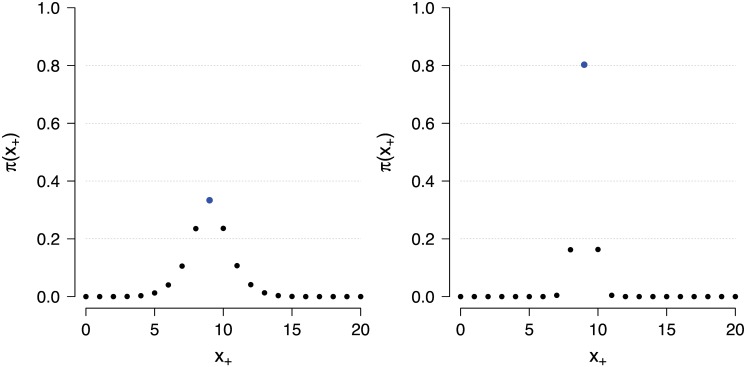
A mixing distribution $\pi (x_{+}^{*})$ for SVE with oversampling. The distribution of transition kernels, i.e., $\pi (x_{+}^{*})$, for sampling from the posterior *π*(*θ* ∣ *x*_+_ = 9) when choosing the best one out of *m* = 5 generated proposals (left panel) and *m* = 20 generated proposals (right panel). In this example the acceptance rate was equal to 75% when generating *m* = 5 proposals and equal to 95% when generating *m* = 20 proposals.

**Fig 3:**
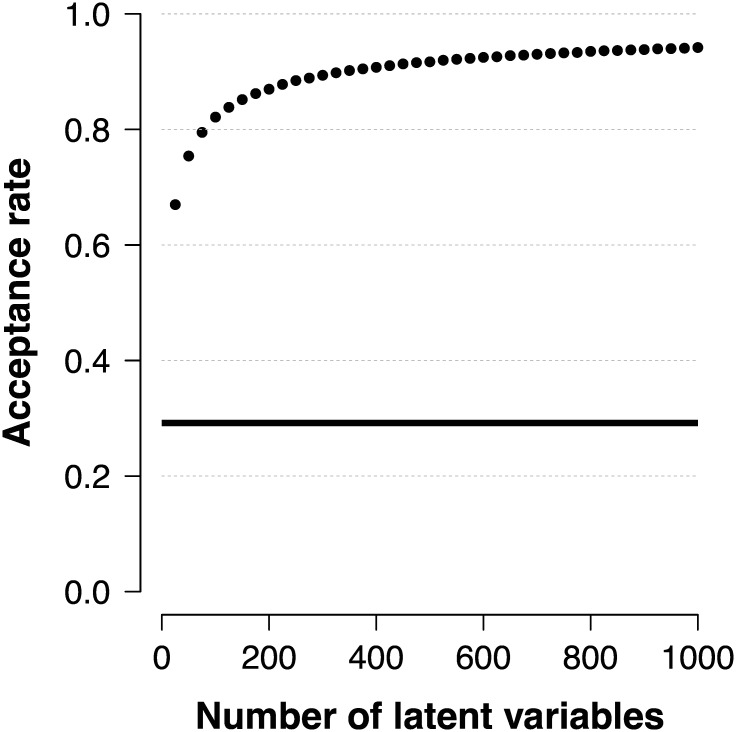
Acceptance rates of the original SVE and SVE with matching. The average proportion of accepted points when simultaneously sampling from *n* target distributions *π*(*θ* ∣ *x*_+_) in the original SVE algorithm (solid line) and the proposal matching procedure (points).

**Fig 4:**
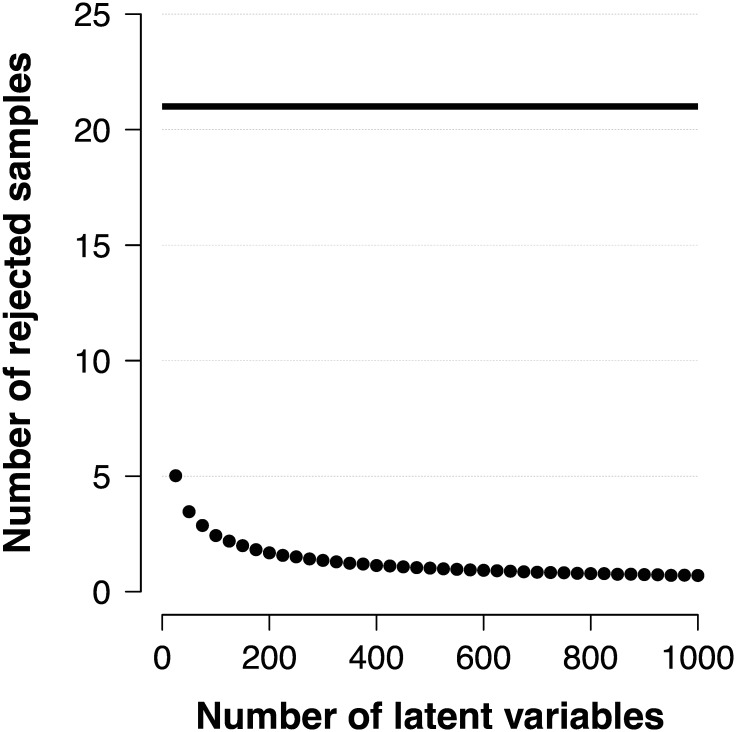
Number of rejected samples for the rejection algorithms. The average number of rejected samples per posterior *π*(*θ* ∣ *x*_+_) out of *n* target distributions in the original Rejection algorithm (solid line) and when rejected values are recycled among the target distributions (points).

**Fig 5:**
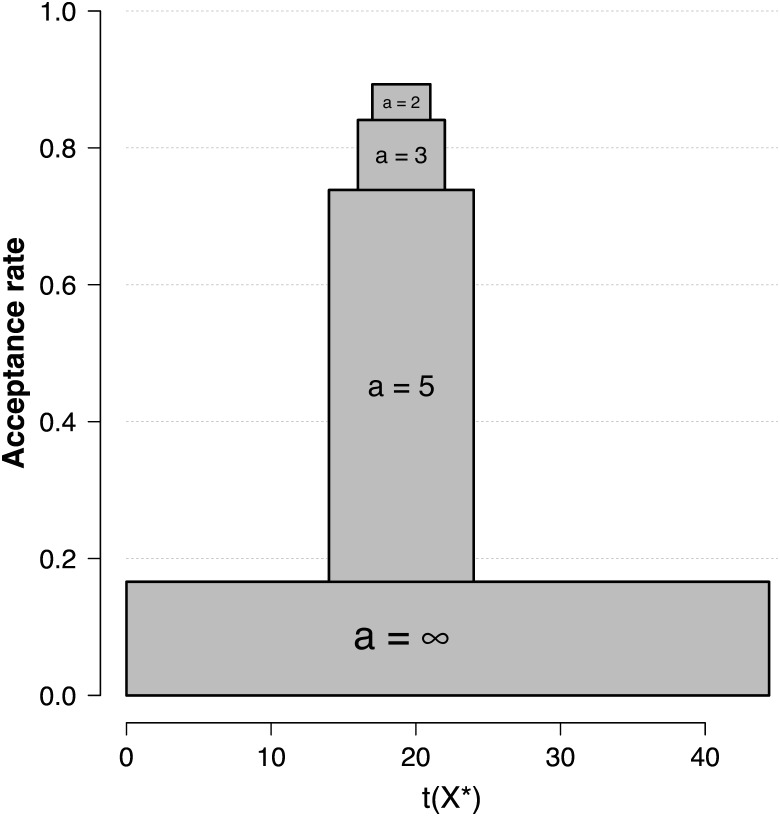
Acceptance rates for the SVE algorithm with different bin sizes *a*. The average proportion of accepted points when proposals are generated until *t*(x*) falls in the range (*t*(*x*) − *a*, *t*(*x*) + *a*) using *a* ∈ {∞, 5, 3, 2}. The gray bars reflect both the range (left and right endpoints) and the proportion of accepted points (top).

**Fig 6:**
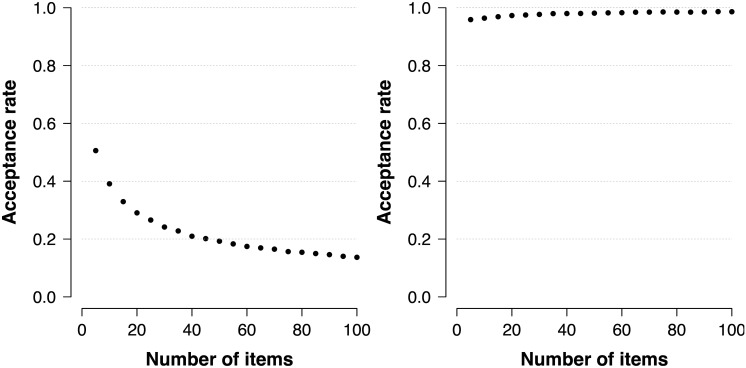
Acceptance rates for the original SVE algorithm and SVE using $\pi (w^{*}|y_{+}^{*})$ as proposal. The average acceptance rate for sampling from posterior distributions *π*(*θ* ∣ *x*_+_) when using the original SVE algorithm (left panel) and when using the proposal distribution $\pi (w^{*}|y_{+}^{*}=x_{+})$ (right panel).

Our secondary goal is to introduce the SVE algorithm as a general purpose method that makes Bayesian inference simple, even for relatively complicated models. As the SVE algorithm does not require much more than the ability to generate data from the statistical model, we believe that it is a practical tool for applied researchers and also serves as a simple introduction to Markov chain Monte Carlo methods for students novel to the field. The extensions that we propose in this paper also fit these two purposes in that they are simple and intuitive extensions of the original SVE approach. To illustrate the original SVE approach and our proposed extensions, we have included annotated R [19] code as supporting information. Specifically, S1–S6 Scripts can be used to reproduce our results (i.e., Figs 1–6), and S7 Script contains the original SVE algorithm and our proposed algorithms in isolation.

Even though we will specifically focus on models in the Exponential Family, we note that our approach also applies to other models by replacing the sufficient statistic with an auxiliary statistic to relate generated data to a parameter. In general one often has a good idea how data and parameters are related, such that it is simple to find efficient auxiliary statistics, an idea that is regularly exploited in Approximate Bayesian Computation [20–22].

Clearly, the main drawback of our approach is the assumption that one is capable of simulating data from the model. That is, we assume that routines to sample (directly) from *π*(*x* ∣ *θ*) and *π*(*θ*) are available. For most models efficient sampling routines are readily available in standard statistical software such as R [19], or can be constructed using general procedures [23, 24]. Extensions of the SVE algorithm where data are sampled using a Markov chain have also been considered [25, 26], and although not investigated here, we anticipate that our approach also extends in this direction. The more general notion is this: if the problem of simulating data is solved, the SVE algorithm turns data simulation into parameter estimation by producing draws from the posterior distribution.

## 2 Methods

### 2.1 A Mixture of Transition Kernels

The factorization in Eq (1) reveals that by first sampling a parameter value θ* ∼ *π*(*θ*) and then data x* ∼ *π*(*x* ∣ θ*), with probability (density) *π*(x*) we have generated θ* from the proposal *π*(*θ* ∣ x*). With the acceptance probabilities *ϕ* defined as in Eq (2), we have that each generated proposal *π*(*θ* ∣ x*) corresponds to a unique Metropolis transition kernel, i.e., a transition probability distribution with density:

$$\pi \left(\theta_{t+1}|\theta_{t}\text{,}\;x\text{,}\;x^{*}\right)=\left\{\begin{array}{cc} \pi \left(\theta^{*}|x^{*}\right)\;\phi \left(\theta_{t}\text{,}\;\theta^{*}|x\text{,}\;x^{*}\right) & \;\text{if}\;\theta_{t}\neq \theta_{t+1}\\ 1-\int_{\Omega_{\theta }}\pi \left(\theta^{*}|x^{*}\right)\;\phi \left(\theta_{t}\text{,}\;\theta^{*}|x\text{,}\;x^{*}\right)\;\text{d}\theta^{*} & \;\text{if}\;\theta_{t}=\theta_{t+1} \end{array}\right.$$

for which the posterior *π*(*θ* ∣ *x*) is the invariant distribution:

$$\pi \left(\theta |x\right)=\int_{\Omega_{\theta }}\pi \left(\theta |\theta^{\prime }\text{,}\;x^{*}\text{,}\;x\right)\;\pi \left(\theta^{\prime }|x\right)\;\text{d}\theta^{\prime }.$$

Since each Metropolis kernel in the SVE algorithm has the same invariant distribution, so does their mixture [4, 17]:

$$\pi \left(\theta^{*}|x\right)=\int_{\Omega_{x^{*}}}\int_{\Omega_{\theta }}\pi \left(\theta^{*}|\theta \text{,}\;x^{*}\text{,}\;x\right)\;\pi \left(\theta |x\right)\;\pi \left(x^{*}\right)\;\text{d}\theta \;\text{d}x^{*},$$

where the integration is over all possible generated datasets Ω_x*_. This formulation shows that the inefficiency of the SVE algorithm is caused by the generation of kernels that have a low probability of making a move, i.e., kernels corresponding to statistics *t*(x*) that are far from the observed statistic *t*(*x*) (c.f. Eq (3)).

To illustrate, consider a simple example from educational measurement; the Rasch model [27]. The Rasch model describes the distribution of a pupil’s item responses as a function of an ability parameter *θ*, and a difficulty parameter *δ* for each of *k* items:

$$\pi \left(x|\theta \right)=\frac{\exp\left\{\sum_{i}x_{i}\theta -\sum_{i}x_{i}\delta_{i}\right\}}{\prod_{i}\left(1+\exp\left\{\theta -\delta_{i}\right\}\right)},$$

where *X* = 1 denotes a correct response and *X* = 0 an incorrect response. The test score *x*_+_ = ∑_*i*_ *x*_*i*_ is sufficient for *θ* such that its posterior depends on the data only through the test score [28], and the mixture ranges over the *k* + 1 possible test scores $\Omega_{x_{+}^{*}}=\left\{0,1,...,k\right\}$:

$$\pi \left(\theta^{*}|x_{+}\right)=\sum_{\Omega_{x_{+}^{*}}}\int_{\Omega_{\theta }}\pi \left(\theta^{*}|\theta \text{,}\;x_{+}^{*}\text{,}\;x_{+}\right)\;\pi \left(\theta |x_{+}\right)\;\text{d}\theta \;\pi \left(x_{+}^{*}\right).$$

The mixing distribution (i.e., test score distribution) *π*(*x*_+_) is shown in Fig 1 for a test consisting of *k* = 20 items, and confirms that it gives much weight to kernels corresponding to values of $t\left(x^{*}\right)=x_{+}^{*}$ that are far from the observed value *t*(*x*) = *x*_+_. For instance, for a pupil with test score *t*(*x*) = *x*_+_ = 9, say, the probability to generate a kernel corresponding to values $t\left(x^{*}\right)=x_{+}^{*}$, with $|x_{+}^{*}-x_{+}|>4$, is approximately 35% in this example (see S1 Script).

## 3 Results

### 3.1 Oversampling for Single Parameter Updates

We wish to assign more weight to kernels with a high probability of accepting a move, while preserving the correct invariant distribution *π*(*θ* ∣ *x*). A simple way to achieve this is to generate *m* ≥ 1 proposed points and then select the one that yielded a sufficient statistic *t*(x*) most similar to *t*(*x*) (c.f. Eq (3)), where *m* = 1 results in the original SVE algorithm. Just as in the original SVE algorithm we have that the posterior distribution *π*(*θ* ∣ x*) is the invariant distribution. Fig 2 illustrates the improvement of this procedure in our Rasch model example for a test score *x*_+_ = 9; improving the probability of directly generating from the target (where $x_{+}^{*}=x_{+}$) from 0.1 to about 0.4 with *m* = 5 samples and about 0.8 with *m* = 20 samples. Even when no direct sample was produced, the proposal distributions became increasingly more similar to the target distribution, thus increasing the overall probability of making a move.

In the application above, we have used functions *t*() of the observed *x* and simulated data x* to select a proposal distribution (i.e., a transition kernel). In practice, however, we might have more information available that informs the selection of good proposal distributions, and one can use functions *f*() that incorporate this information. In the next section we illustrate such a function that incorporates, for instance, covariates that are used in the statistical model. Observe that we do not use the current state of the parameter, *θ*′, or the proposed point, θ*, to select a proposal distribution. This ensures that the posterior distribution *π*(*θ* ∣ *x*) remains the correct invariant distribution of the Markov chain.

Fig 2 reveals that using the sufficient statistic we are able to select statistically more efficient proposals as *m* increases. This follows from inspecting the acceptance probability in Eq (3), and observing that the statistically more efficient proposals are those for which |*t*(x*) − *t*(*x*)| is at a minimum, and furthermore that the minimum min_*m*_ {|*t*(x*) − *t*(*x*)|} over *m* proposals is non-increasing with *m*, i.e.,

$$\min_{m}\left\{|t\left(x^{*}\right)-t\left(x\right)|\right\}\geq \min_{m+1}\left\{|t\left(x^{*}\right)-t\left(x\right)|\right\}.$$

It is important to note that the *m* proposals can be generated in parallel so that the oversampling of proposals need not increase the computational burden. However, only one of the *m* proposals is subsequently accepted by the Markov chain. As we will see next, all generated proposals can be put to good use when simultaneously sampling from more than one target distribution.

### 3.2 Matching for Multiple Parameter Updates

With the commonly assumed conditional independence of observations in hierarchical models, we have independent posterior distributions for each of *n* random effects (or latent variables) [28]:

$$\pi \left(\theta^{*}|x\right)=\prod_{p=1}^{n}\pi \left(\theta_{p}|x_{p}\right).$$

Since proposals are also independently generated, it is convenient to consider the joint application of *n* independent SVE kernels:

$$\pi \left(\theta^{*}|x\right)=\int_{\Omega_{x}}\prod_{p=1}^{n}\int_{\Omega_{\theta }}\pi \left(\theta_{p}^{*}|\theta \text{,}\;x_{p}^{*}\text{,}\;x_{p}\right)\;\pi \left(\theta |x_{p}\right)\;\text{d}\theta \;\pi \left(x^{*}\right)\;\text{d}x^{*},$$

where the original SVE algorithm has

$$\pi \left(x_{p}^{*}|x_{\backslash p}^{*}\right)=\pi \left(x_{p}^{*}|x_{1}^{*},\cdots ,x_{p-1}^{*},x_{p+1}^{*},\cdots ,x_{n}^{*}\right)=\pi \left(x_{p}^{*}\right),$$

due to independence. We wish to modify *π*(x*) such that each component $\pi \left(x_{p}^{*}\right)=\int \pi \left(x^{*}\right)\;\text{d}x_{\backslash p}^{*}$ assigns more weight to kernels with a high probability of accepting a move.

Similar to our oversampling procedure we can generate *m* ≥ 1 proposals and assign each of the generated proposals to a target distribution. Here, we choose the number of generated proposals to be equal to the number of target distributions, which implies that we simply rearrange the *m* = *n* generated proposals. We wish to rearrange the proposals such that each of the *n* kernels has a high probability of accepting the proposed point; i.e., match proposals to targets such that for each target distribution the proposal statistic *t*(x*) is close to the observed statistic. However, even for relatively small values of *n* it becomes expensive to search the *n*! possible arrangements for the statistically most efficient one, which suggests to use a simple rule to automatically choose an arrangement given a generated dataset x*.

We illustrate such a simple rule with sampling from the posterior distribution of *n* ability parameters in the Rasch model;

$$\pi \left(\theta |x_{+}\right)=\prod_{p=1}^{n}\pi \left(\theta_{p}|x_{p+}\right).$$

We order the posterior distributions based on the corresponding test scores; those corresponding to a low test score are placed first whereas those corresponding to a high test score are placed last. Next, we generate *m* = *n* proposal distributions which are ordered in the same way as the target distributions; those corresponding to a low generated test score are placed first and those corresponding to a high generated test score are placed last. It is clear that the first proposal is likely to be a good proposal for the first target distribution, the second proposal for the second target distribution, and so on. That this procedure improves the statistical efficiency is shown in Fig 3. The solid horizontal line in Fig 3 shows the average acceptance rate of the original SVE algorithm applied separately to each of the *n* latent variables, the efficiency of which is independent of *n*, and the points refer to the acceptance rate using our kernel matching procedure. Even with as little as *n* = 25 latent variables there is a substantial improvement to the statistical efficiency when the proposals are matched, with an average acceptance rate of 29% in the original SVE algorithm and 67% when the proposals are matched. Moreover, Fig 3 reveals that the statistical efficiency continues to improve as *n* increases.

Fig 3 reveals that if *t*(*x*) is sufficient for *θ*, we have a good way to match proposals to targets and as *n* becomes sufficiently large, each kernel tends to make a move such that we sample approximately i.i.d. from each of the *n* posteriors. We note that the proposals can be generated and subsequently accepted in parallel. The only non-parallizable part of the procedure is in matching the proposals, although one can find clever ways to do this. Sorting the statistics *t*(x*) (posterior distributions) is of an order of complexity that is often usually *n* log *n* but at most *n*^2^, which compares favorably to the order of complexity *n*! that is needed to find the statistically most efficient rearrangement.

Sampling-unit specific prior distributions *π*_*p*_(*θ*) are easily handled by incorporating the information encoded in the prior distributions, such as covariates, in matching the proposals. Since this information is also encoded in the mixing probabilities, it is available for matching proposals to target distributions. The only difference is that when a point drawn from *π*_*q*_(*θ*) is proposed to a posterior with prior density *π*_*p*_(*θ*), *p* ≠ *q*, the prior distributions do not cancel from the expression in Eq (3), and we accept θ* with probability:

$$\min\left(1\text{,}\;\exp\left\{\;\left(\theta^{*}-\theta^{\prime }\right)\cdot \left(t\left(x\right)-t\left(x^{*}\right)\right)\;\right\}\times \frac{\pi_{p}\left(\theta^{*}\right)\;\pi_{q}\left(\theta^{\prime }\right)}{\pi_{p}\left(\theta^{\prime }\right)\;\pi_{q}\left(\theta^{*}\right)}\right),$$

ensuring that *π*(*θ* ∣ *x*) ∝ *π*(*x* ∣ *θ*)*π*_*p*_(*θ*) remains the invariant. For most prior distributions, the ratio of prior distributions is easily computed as many parts cancel in the expression.

To illustrate, consider a latent regression model in which each of *n* abilities *θ* is assigned a unique prior distribution:

$$\theta_{p}|y_{p}∼\pi_{p}\left(\theta \right)=N\left(y_{p}^{T}\beta \text{,}\;\sigma^{2}\right),$$

where *y*_*p*_ constitutes a covariate vector for person *p* and *β* a vector of regression weights. Assuming that a point drawn from *π*_*q*_(*θ*) is proposed to a posterior with prior density *π*_*p*_(*θ*), *p* ≠ *q*, we consequently accept θ* with probability:

$$\min\left(1\text{,}\;\exp\{\;\left(\theta^{*}-\theta^{\prime }\right)\cdot \left(\left[x_{p+}+y_{p}^{T}\beta /\sigma^{2}\right]-\left[x_{q+}^{*}+y_{q}^{T}\beta /\sigma^{2}\right]\right)\;\}\right),$$

which also reveals that it is opportune to use the statistic $x_{p+}+y_{p}^{T}\beta /\sigma^{2}$ to match proposals to targets.

### 3.3 A Rejection Algorithm

When *t*(X*) is a discrete random variable, a simple Rejection procedure is to generate proposals until an exact match *t*(x*) = *t*(*x*) is produced [2]. The matching of kernels points to an extension of the Rejection algorithm for sampling from *n* ≥ 1 posteriors. Consider sampling from the posterior distribution of *n* ability parameters in the Rasch model using a common prior. There are at most *k* + 1 different posterior distributions to sample from; one for every possible test score. Let $n_{x}{}_{{}_{+}}$ denote the number of observations for a test score *x*_+_. We generate a proposal corresponding to a test score $x_{+}^{*}$; if $n_{x_{+}^{*}}>0$ we retain the proposed point and decrease $n_{x_{+}^{*}}$ by one, otherwise we reject the proposed point. This procedure is repeated until $n_{x}{}_{{}_{+}}=0$ for each possible test score, after which the generated values can be assigned to the target distributions. Note that this simplifies to the original rejection algorithm when $n=\sum_{x_{+}}n_{x}{}_{{}_{+}}=1$.

Fig 4 shows that recycling the otherwise wasted proposals can significantly improve the computational efficiency (reduce the order of complexity). The solid horizontal line shows the average number of proposals that need to be generated when the original rejection algorithm is applied separately to each of *n* latent variables, the efficiency of which is independent of *n*, and the points show the average number of proposals that need to be generated when the proposals are recycled. Most significant is the reduction of the computational expense when samples are required from increasingly larger numbers of target distributions. When *n* becomes sufficiently large, only *n* proposals need to be generated to sample once from each of the *n* target distributions.

### 3.4 Binning the Statistics

The rejection algorithm is unsuited for applications in which *t*(*X*) is a continuous random variable or a discrete random variable with many possible realizations. Even though repeated sampling does not guarantee an exact match, oversampling revealed that we do continue to produce better proposals. In general, a good proposal is one for which the statistic *t*(x*) is “sufficiently close” to *t*(*x*); i.e., *t*(x*) is in some range $T_{a}=\left(t\left(x\right)-a\text{,}\;t\left(x\right)+a\right)$, with *a* > 0. This suggests that we generate proposals until $t\left(x^{*}\right)\in T_{a}$, with the value of *a* controlling the quality of our proposals.

To illustrate, consider a simple extension of our Rasch model known as the two-parameter logistic model. The two-parameter logistic model describes the distribution of a pupil’s item responses as a function of the ability parameter *θ* and an item discrimination *α*_*i*_ and difficulty *δ*_*i*_ for each of *k* items:

$$\pi \left(x|\theta \right)=\frac{\exp\{\sum_{i}\alpha_{i}x_{i}\theta -\sum_{i}x_{i}\delta_{i}\}}{\prod_{i}\left(1+\exp\left\{\alpha_{i}\theta -\delta_{i}\right\}\right)},$$

where the weighted test score *t*(*x*) = ∑_*i*_ *α*_*i*_ *x*_*i*_ is sufficient for *θ*. Since the discrimination parameters *α*_*i*_ are real-valued (typically positive) we have that *t*(*X*) is a discrete random variable with 2*^k^* possible realizations, one for every possible vector of item scores.

We consider sampling from a posterior *π*(*θ* ∣ *t*(*x*)) for a weighted test score *t*(*x*) ≈ 19 (*x*_+_ = 9) based on a *k* = 20 item test with discriminations *α*_*i*_ that are sampled uniformly between 0 and 4. Fig 5 reveals that generating proposals until *t*(x*) falls in a bin $T_{a}$ increases the quality of proposals as a function of *a* (using *a* ∈ {∞, 5, 3, 2}); improving the overall acceptance rate from approximately 17% for *a* = ∞ (the original SVE algorithm) to approximately 74% for *a* = 5.

The idea of generating proposals until the statistic *t*(x*) falls within a certain range $T_{a}$ is closely related to the idea behind the ABC-rejection algorithm, where one simply accepts a proposed point when $t\left(x^{*}\right)\in T_{a}$ [21, 22]. When *a* is “sufficiently small” the proposed point θ* will be drawn from a posterior distribution *π*(*θ* ∣ *t*(x*)) that is “close” to the target distribution *π*(*θ* ∣ *t*(*x*)). For the SVE approach *a* need not be “sufficiently small” as the Metropolis kernel ensures that the correct posterior distribution *π*(*θ* ∣ *x*) is the invariant distribution. It is clear that decreasing the value of *a* implies higher acceptance rates, but also that more samples are required to produce a value *t*(x*) in $T_{a}$, on average. However, when there are multiple target posterior distributions one could bin the observed statistics into *m* non-overlapping ranges, and apply recycling to the *m* bins to reduce the number of samples that need to be produced.

### 3.5 A Data Augmentation Procedure

Matching and oversampling use auxiliary- or sufficient statistics *t*(x*) to choose more efficient proposals. Marsman, Maris, Bechger and Glas [29] generalized this approach by making clever use of the augmented variables that are often used to sample from *π*(*x* ∣ *θ*). For example, logistic random variables are commonly used to sample item scores in the Rasch model:

$$\pi \left(X=1|\theta \text{,}\;\delta \right)=\int_{-\infty }^{\theta -\delta }\frac{\exp(-z)}{{\left(1+\exp\left(-z\right)\right)}^{2}}\;\text{d}z.$$ (4)

Although Gibbs samplers are frequently used to sample from the augmented posteriors *π*(*z*, *θ* ∣ *x*), such procedures tend to converge slowly. The solution to this problem is to only use the augmented variables to generate proposals from a slightly different model that, when used in an independence chain (i.e., the SVE algorithm), does not suffer from this slow convergence.

Consider the distribution of **w** = {*z*_1_, …, *z_k_*, *θ*}; the joint distribution of the augmented variables and the parameter. We have that the conditional distribution $\pi (w_{k+1}^{*}|w_{\backslash k+1}^{*})$ corresponds to a unique posterior distribution $\pi (w_{k+1}^{*}=\theta^{*}|x^{*})$, where x* is a function of w* defined through relations as Eq (4) (i.e., $x_{i}^{*}=1$ if $w_{i}^{*}<w_{k+1}^{*}$, and 0 otherwise). Note that this relation can also be used to define posteriors $\pi (w_{i}^{*}|y^{*})$ for each of the *k* remaining conditional distributions $\pi (w_{i}^{*}|w_{\backslash i}^{*})$. The difference between the posterior $\pi (w_{k+1}^{*}|x^{*})$ and $\pi (w_{i}^{*}|y^{*})$ is that *w*_*k*+1_ = *θ* is used as the augmented variable in $\pi (w_{i}^{*}|y^{*})$ and *w*_*i*_ = *z*_*i*_ is the proposed point, whereas *w*_*k*+1_ = *θ* is the proposed point in $\pi (w_{k+1}^{*}|x^{*})$ and *w*_*i*_ = *z*_*i*_ is used as the augmented variable to generate $x_{i}^{*}$. This means that $\pi (w_{i}^{*}|y^{*})$ is a posterior distribution that corresponds to a slightly different model that uses the same model components, but where one of the likelihood functions switched places with the prior distribution (see Ref. [29] for more details). What makes this approach interesting is that from generating a single data vector we obtain *k* + 1 proposal distributions and we can choose the statistically most efficient one.

Fig 6 illustrates the approach with sampling from posterior distributions *π*(*θ* ∣ *x*_+_) in the Rasch model using $\pi (w^{*}|y_{+}^{*}=x_{+})$ as the proposal distribution (right panel). Note that the procedure is statistically efficient and further improves when more observations become available; even though the posteriors become more concentrated. Also shown in Fig 6 is the original SVE approach (left panel), which becomes less efficient as the number of observations increases.

The procedure applies also to Logistic regression models, and, when the augmented variables have a non-logistic distribution, for instance a normal distribution, we obtain other Bernoulli regression models, such as probit regression [30]. Marsman et al. [29] used the procedure to estimate Ising network models using a full-data-information procedure, utilizing a latent variable expression of the Ising model, where the conditional distribution *π*(*x* ∣ *θ*) was found to be a multidimensional two-parameter logistic model [31].

Exponential Family models are closed under conditioning, that is, *π*(*x* ∣ *θ*, *x* ∈ *ω* ⊂ Ω_*x*_) is also in the Exponential Family. In this manner, we find that generating responses to two Rasch items corresponds to a three category Partial Credit Model [32] whenever

$$\left(x_{1},x_{2}\right)\in \left\{\left(0,0\right),\left(1,0\right),\left(1,1\right)\right\}\subset \Omega_{x},$$

and the procedure similarly extends to such situations. In principle, this procedure can be used to generate other models, such as multinomial logit models [33], and extends the framework of Marsman et al. [29] to Potts network models.

## 4 Discussion

With the SVE algorithm a powerful yet simple idea was introduced; any model that can be simulated can be estimated. Based on a mixture of Metropolis kernels representation we have built upon the idea introduced with the original SVE algorithm and suggested several approaches that produce significant improvements to the convergence and autocorrelation of the Markov chain. To keep things simple, we have focused explicitly on statistical models *π*(*x* ∣ *θ*) that are in the Exponential Family. However, the approaches that we have proposed in this paper are more generally applicable and simple to implement.

## Supporting Information

S1 Script Annotated R-Code for Fig 1. (TXT)

S2 Script Annotated R-Code for Fig 2. (TXT)

S3 Script Annotated R-Code for Fig 3. (TXT)

S4 Script Annotated R-Code for Fig 4. (TXT)

S5 Script Annotated R-Code for Fig 5. (TXT)

S6 Script Annotated R-Code for Fig 6. (TXT)

S7 Script Annotated R-Code with Proposed Procedures. (TXT)
